# Measuring Multiple Text Integration: A Review

**DOI:** 10.3389/fpsyg.2018.02294

**Published:** 2018-11-29

**Authors:** Liron Primor, Tami Katzir

**Affiliations:** Department of Learning Disabilities, University of Haifa, Haifa, Israel

**Keywords:** text integration, reading comprehension, assessment methods, adults population, synthesis

## Abstract

Multiple text integration is an important skill in modern society, required in heterogeneous situations, across many disciplines and in daily life. It is a complex skill that builds on bottom-up and top-down processes (Britt and Rouet, 2012). As a complex skill it has been measured in the literature using different techniques. To date, the different ways in which researchers have defined and operationalized the term have not been reviewed. Therefore, the aim of this paper is to review how multiple text integration has been theoretically and empirically assessed. The current paper reviews which texts were typically used, which aspects of integration were assessed, and with which scoring rubrics. Finally, we propose that despite the diverse use of tasks, important features of multiple text integration are missing from current research.

## Introduction

The internet era has changed the very nature of reading. Today, in the click of a mouse, readers are exposed to multiple sources of information on the same topic in personal, academic, occupational, and social contexts (List and Alexander, 2017). This might include reading articles and blogs in order to reach decisions concerning recreational activities, investments, and health, or reading articles for academic purposes. Reading online material almost always involves multiple texts in the form of hyperlinks and comments (Eshet-Alkalai, 2004; Liu et al., 2008; Goldman and Scardamalia, 2013). For this reason, Goldman et al. (2012) argued that multiple text reading is the norm rather than the exception.

Generating a coherent representation based on multiple texts is a demanding task that builds on numerous top-down and bottom-up processes (Rouet, 2006; Britt and Rouet, 2012); in order to construct a coherent and complete representation of multiple texts, readers first need to efficiently process each text separately and build a situation model in the context of a certain reading goal (Kintsch, 1988). Readers also need to evaluate the reliability and relevance of each text (Richter, 2011). Furthermore, they must construct a model of the relationships among the texts. They do so based on several processes: they link details and ideas between the texts (Rouet and Britt, 2011); they compare and contrast information across texts, notice inconsistencies, and decide how to deal with them; finally, they organize the various details and ideas into a coherent representation (Goldman et al., 2012).

Integration of multiple texts is a complex procedure that can result in different types of integration. Texts can be integrated based on their contents, linguistic features, rhetorical aspects, or information external to the text, such as the source or context of the writing. Different logical relationships can be formed, such as cause and effect, chronology, hierarchy, etc. Finally, readers can integrate segments of information that are explicitly stated, or implicit ideas that are deduced from the texts. Integration will also be influenced by text and task features (Snow, 2002).

Whereas research on single text comprehension deals with types and levels of reading comprehension and their correspondence to different assessment methods, a similar discussion is currently missing from multiple text research. For example, single text reading comprehension studies compared reading comprehension assessment methods, and conclusions were reached regarding what skills are tapped by the various measures (e.g., Cutting and Scarborough, 2006; Keenan et al., 2008; Keenan, 2012). Theoretical models have also addressed the notion that there are different levels of comprehension, tapped by various assessment methods (Kintsch, 1988; McNamara et al., 2015).

Multiple text integration (MTI) has been assessed with different types of tasks based on expressive, receptive, and think aloud procedures. Even while using similar tasks, the scoring rubric utilized in the literature varies (e.g., Barzilai et al., 2018). Nevertheless, discussions of types, levels of integration, and comparisons of assessment methods are scarce. Therefore, the aim of this paper is to examine which aspects of MTI have been studied, by carefully mapping MTI tasks and theoretical models. We will begin by discerning the nature of MTI, and how this concept was defined and assessed in the literature. We will review MTI assessment methods and compare them on the basis of the texts, tasks, and assessment rubrics. Finally, we will point to important gaps in the current literature and suggest future directions of research.

## Theoretical Models of MTI

The first studies that focused on the integration of multiple texts were conducted during the 1990s by Wineburg (1991, 1998), and investigated differences between expert and novice reading of multiple texts in the history discipline. Wineburg (1991, 1998) work focused on the field of history, where scholars must integrate and reconcile conflicts between different sources. Results of these studies pointed to major differences in reading strategies between experts and novices. The findings suggested that only experts use strategies such as sourcing, corroboration, and contextualization. Expert readers paid attention to the source and context and looked for inconsistencies across documents, whereas novices did not apply these strategies and were not able to deal with conflicts. Following these works, more and more studies suggested that MTI is a challenging task even for college students (e.g., Segev-Miller, 2007). Following Wineburg (1991, 1998) work in history, the research expanded to study MTI in more disciplines and new contexts, and examined a variety of aspects of engagement with multiple texts, such as use of sources (e.g., Strømsø et al., 2010), individual differences (e.g., Barzilai and Strømsø, 2018), task characteristics (e.g., Gil et al., 2010a,b), and how to promote integration (e.g., Barzilai et al., 2018).

In addition, theoretical models were developed to describe the mental structures that readers generate when deeply engaging with multiple texts (DMF, Britt et al., 1999; Perfetti et al., 1999; Rouet, 2006) and the cognitive procedures that readers undergo (MD-TRACE; Rouet and Britt, 2011; Goldman et al., 2012). Contextual factors (RESOLV, Rouet et al., 2017; MD-TRACE; Rouet and Britt, 2011) and individual differences in MTI were also discussed (CAEM; List and Alexander, 2015, 2017, MD-TRACE; Rouet and Britt, 2011; Britt and Rouet, 2012). In addition, the role of contradictions between texts (Braasch et al., 2012; Braasch and Bråten, 2017) and the interaction between readers’ prior knowledge and texts’ contents were addressed (Richter and Maier, 2017). Different frameworks investigated how readers derive meaning from multiple texts and use different terminology to describe it (Barzilai et al., 2018).

In order to clarify the various terms, MTC (multiple text comprehension) is used to describe readers’ engagement with multiple texts, and includes a variety of processes and abilities such as understanding the literal meaning of the texts, noticing sources and differences between them, etc. MTI (multiple text integration) describes the specific act of linking pieces of information from various texts, which is a subprocess or component of MTC. The current review includes models stemming from reading research, dealing with MTC and MTI.

Bråten et al. (2013a, pp. 322–323) defined multiple text comprehension (MTC) as the “building of a coherent mental representation of an issue from the contents of multiple text that deal with the same issue from different perspectives.” List and Alexander (2017, p. 143) used a similar definition, but one that emphasized the processes involved rather than the product of comprehension: “MTC refers to the processes and behaviors whereby students make sense of complex topics or issues based on information presented not within a single source but rather across multiple texts.” MTC has also been referred to as “multiple documents literacy” and “multiple sources comprehension.”

Goldman et al. (2012, 2013) proposed a model that breaks down MTC into several subcomponents and offers a specific definition of MTI. The subcomponents include: gathering resources; sourcing and selecting resources; analyzing, synthesizing, and integrating information within and across sources; applying information to accomplish the task; and evaluating processes and products. According to Goldman et al. (2013), analysis, synthesis, and integration operate across sources as well as within sources (see also Perfetti et al., 1999). *Analysis* is the process of sorting out the information relevant to the inquiry task, since not all the material is relevant for the specific task. *Synthesis* across multiple texts is defined as an inferential reasoning process that compares and contrasts contents in order to determine the relationship between the various pieces of information (e.g., complementary, overlapping, or redundant). Finally, integration “involves organizing the outcomes of analysis and synthesis processes to form the integrated model” (p. 185).

The documents model framework (DMF; Britt et al., 1999; Perfetti et al., 1999; Rouet, 2006), the first theoretical model to account for MTC, did not explicitly define integration, but rather described the types of links that are formed during MTC. The model outlined the mental structures that readers generate in order to represent source information and to assemble heterogeneous and sometimes conflicting document information to create an *integrated mental model* (Britt et al., 2013). The integrated mental model (originally called the *intertext model*) is a product of two types of links: links between the information represented in each text (referred to as *Document node*), and links between contents and sources (called intertext Links). Connections between contents are made across the different levels of presentation, so that a reader might notice similarities or differences in surface structure, text base, or situation model (Britt and Sommer, 2004).

Britt et al. (2013) explained that the documents model assumes that a document is an “entity,” which means that readers do not encounter an isolated text but rather a text that was written by an author, with certain values and motives, within a specific context. The various features of the source, such as the identity of the author and his or her aim in writing, are important for the selection, evaluation, and synthesis of the various texts. In addition, Perfetti et al. (1999) point out that factors such as type of task and reading goal may influence the quality of the integrated situation model and of the intertext model.

The MD-TRACE model (Rouet and Britt, 2011; Britt and Rouet, 2012) expands the DMF to include not only the mental structures the reader generates, but also a description of specific processes, products, and resources needed to complete tasks that involve multiple texts. One of the steps involved is *text processing* that leads to the next step, *formation or update of the documents model*. Yet, the exact procedures that take place in this step are not elaborated.

Two recently proposed models discuss the role of inconsistencies and contradictions between texts, or between texts and prior knowledge and beliefs in MTC. These models thus imply that MTI is based on processes of comparison between types of information. The DIS-C model (Braasch et al., 2012; Braasch and Bråten, 2017) “provides a detailed description of processes that occur when reading-to-understand controversial messages presented by multiple information sources” (Braasch and Bråten, 2017; p. 2). The model builds on both single text discourse comprehension theories as well as on MTC models and focuses on contradictions between texts or between previous knowledge and the current text, as conditions that stimulate a deeper processing and consideration of information sources. These conflicts are assumed to promote attention to source information (who the author is, context of writing, where it was published, etc.) as means of resolving the conflict.

Similarly, Richter and Maier (2017) discuss the role of prior knowledge and beliefs in comprehending multiple texts that are consistent or inconsistent with readers’ prior beliefs. According to their two-step model, readers detect text-belief inconsistencies through a routine process of *validation*, or *epistemic monitoring* of incoming text information for internal consistency and plausibility (Richter, 2011). In the next step, factors such as motivation and reading goal affect the extent of strategic effortful processing of the inconsistent information.

In a paper dealing specifically with MTI, Cerdán Otero (2005, p. 25) suggested the following definition of MTI: “A mental process that connects different units of information into the reader’s mind.” She further proposed that this process is the product of two strategies: The first is corroboration, which means comparing and contrasting information from several documents in order to identify unique pieces of information, contradictions, and overlaps. The second is the reinstatement-and-integration strategy proposed by Mannes (1994); Mannes and Hoyes (1996). According to this strategy, when reading multiple texts, sets of prepositions that were read before are reinstated when relevant prepositions are read in another text, on the basis of similarity. This allows integration of the two sets of prepositions by means of inference making and elaborations. Cerdán Otero (2005) also points out that this is an active, effortful, and time-consuming process rather than an automatic process, and that it also depends on the characteristics of the task and on the relationship between the texts, which may favor or limit such active processing.

In conclusion, MTC has been conceptualized by a variety of models, while only some of them have specifically and directly discussed MTI. Two definitions of integration were presented, sharing the core idea that integration is the act of linking and synthesizing pieces of information. Yet, the nature of these links was not specified. Links between texts are formed on the basis of similarities and differences between pieces of information, a process that is referred to as synthesis (Goldman et al., 2012, 2013), epistemic validation (Richter, 2011), or corroboration (Wineburg, 1991; Cerdán Otero, 2005). This synthesis occurs within the contents of the texts, and between the contents and source information (Perfetti et al., 1999; Britt et al., 2013). Next, we sought to examine how theoretical models of MTI were translated to empirical tasks.

## Goals of the Current Review

The MTC models described above provide a general definition of MTI, suggestions as to the processes and strategies readers employ while engaging with multiple texts, and speculated factors that also take part in this process (such as context, reader, and task characteristics). However, the various theoretical models rarely discuss the type of links formed between texts and how they are represented in the empirical assessment methods used in various studies. We were therefore interested in a thorough review of types of integration tasks and two questions were asked: What types of integration tasks were studied in the empirical research? What types or levels of integration were addressed, based on the nature of the tasks given to readers? To address these questions, we reviewed how integration was assessed across studies, and compared text types, tasks, and assessment rubrics.

## Methods

### Literature Base and Inclusion Criteria

Multiple text integration studies were retrieved by searching peer-reviewed journals published in English in the PsycINFO and ERIC databases. Articles published until 2017 were included.

Following the literature review we chose several keywords and formed the following search string for searching in article titles: (multiple AND text^∗^) OR (multiple AND document^∗^) OR (text^∗^ AND integ^∗^) OR (text^∗^ AND source^∗^). The search was not limited to any time range or population. The search yielded 257 articles from ERIC and 340 from PsychINFO. In addition, we included a classical article that did not come up during the database search (Wineburg, 1991).

Fifty studies met the following inclusion criteria:

(1) Studies that focused on integration between texts. We did not include studies that focused on integration of texts and visual information, integration of words in texts, or integration within single texts. Nevertheless, when integration of texts and pictures was examined along with integration of texts, the study was included (e.g., Wineburg, 1991).
(2) Studies that specifically assessed integration of multiple texts. We did not include studies that used an MTI task in order to measure other constructs (such as attention to sources or memory for conflicts) and did not directly measure MTI.
(3) For reviewing MTI assessment methods we included only empirical studies that specified how MTI was assessed. Studies in which MTI assessment was not sufficiently elaborated were not included. In addition, we found no theoretical studies that reviewed MTI tasks.
(4) We chose to focus on studies conducted in L1.
(5) Studies were written in English, but the research itself was not limited to any language.

### Coding Scheme

In reviewing the literature, we used the following categorizations and coding schemes:

#### Participants

Number of participants and demographic information.

#### Texts

We shortly described the number of texts, their topic, and marked the relationships between them in the following manner: A≠B was used to describe any set of texts that included a contradiction, a conflict, or a disagreement. In this category we included only texts where the conflict was central to the integration. A+B was used to describe texts that were convergent and required adding pieces of information together.

#### Tasks and Assessment Rubrics

We divided the tasks into receptive and expressive tasks, presented in separate tables, and specified the various task types (e.g., essay writing, open ended questions, etc.), since each type of task requires the reader to employ different skills. MTI tasks that required writing or providing oral accounts of comprehension were classified as expressive measures, whereas tasks that demanded marking a correct response were referred to as receptive tasks. In the case of expressive tasks we also included the instructions given to readers (when these were available). As for expressive tasks, a variety of categories were employed in order to assess integration. We examined the scoring technique (e.g., holistic scoring or analyzing smaller units) and the categories used to evaluate the products (e.g., paraphrasing, elaborations, supporting arguments, etc.).

#### Levels of Integration

Next, we endeavored to map the list of evaluation categories used for integration assessment. Following a consultation between the two authors, we divided these into three levels: *selecting information, intertextual relationships*, and *inference making*. The first two categories are similar to the terms analysis and synthesis proposed by Goldman et al. (2013). The first level, *selecting information*, refers to selecting the relevant pieces of information from the various texts and including them in the answers. Goldman et al. (2013) referred to this level as *analysis*. Selection of information means extracting a main idea from a single text in the context of multiple texts, and therefore differs from extracting main ideas from a single text in isolation. We considered categories such as “covering main ideas or arguments” and “referring to sources” (e.g., Blaum et al., 2017) as subsumed under the title of selecting information.

The second category was *generating relationships* *between the texts*. It referred specifically to linking pieces of information extracted from different texts or about sources, and noticing the relationship between them (e.g., complementary, conflicting). Goldman et al. (2013) named this level *synthesis.* We considered categories such as “corroboration” (comparing documents to one another, Wineburg, 1991) and “reconciling conflicts” (e.g., Bråten et al., 2014a), which require the readers to actively form connections between the texts. The third category we used was *inference making*, including what we interpreted as transforming information or adding something new. We considered categories such as “statement including novelty” (e.g., Linderholm et al., 2014) and “using prior knowledge” (e.g., Goldman et al., 2013) to fall under this category.

Each level was divided again to three types: *conceptual, linguistic, and rhetoric* (Segev-Miller, 2007). These terms are borrowed from Segev-Miller (2007), who listed strategies writers employ when synthesizing texts. We found this structure to be useful for pointing to differences between what is measured in expressive versus receptive tasks and for illuminating aspects of text integration. The conceptual level deals with ideas and contents (e.g., covering main ideas or arguments; Blaum et al., 2017). The rhetorical level involves integration as exhibited in the structure of the written text (e.g., relating to sources; Stadtler et al., 2013). Finally, the linguistic level refers to linguistic means that express integrated representation (e.g., using connectives to note relationships between the texts; List and Alexander, 2015).

Mapping assessment categories across the various tasks was a complicated endeavor for several reasons. First, different terms were used and we had to judge whether different terms point to the same concept or, on the contrary, whether identical terms found in several places had different meanings. For example, we judged *rebuttals* and *reconciling conflicts* as referring to the same construct. Second, descriptions of tasks and assessment methods were sometimes not sufficient for us to determine the exact level of integration required. For example, main ideas and arguments can be stated explicitly or, on the contrary, implicitly extracted from the text. Therefore it is possible that in one set of texts extracting main ideas involved higher levels of inference making, while in another text it required only understanding the literal meaning. Third, MTI is a complex task with many underlying processes, such that clear-cut distinctions between these processes are challenging (McNamara et al., 2015). Any disagreements between the two authors in mapping assessments by the categories described above were resolved through discussion and required at least 90% agreement.

Tables 1, 2 present details of text integration studies divided into expressive (Table 1) and receptive tasks (Table 2). In each table, similar tasks are grouped together and are then arranged alphabetically by authors’ names (essay writing, open ended questions, etc.). When several studies used the same MTI assessment method, only one study was fully presented. Other similar studies were mentioned under the task assessment description (“Assessment” rubric), so that the same methodology was presented only once. Table 3 includes results of these studies as well. In total there were 50 studies that examined 61 tasks and used 33 categories to assess them.

**Table 1:** Literature review of expressive integration tasks.

Researchers	Readers	Texts	Task	Assessment
Anmarkrud et al. (2013)	51 students from a large state university in southeast Norway	A≠B Six documents that presented different perspectives on cell phones and potential health risks.	**Essay writing** “You are now going to write a short essay where you judge the health risk of cell phone use. Base your response on the texts that you just read and try to express yourself clearly and elaborate the information—preferably in your own words. Justify your conclusions by referring to the sources you have been working with.”	The essays were coded from 1 to 7 for: • Including a position on the issue • Supporting and opposing reasons • Elaborations • Rebuttals See also: Bråten et al. (2014b); Bråten et al. (2015).

Barzilai and Eshet-Alkalai, 2015	170 Hebrew-speaking students from an Israeli university	A≠B, A+B Four texts, designed as blog posts, dealing with increasing use of seawater desalination in Israel. The four texts were either convergent or conflicting (two conditions).	**Essay writing** “Please write an argument that addresses the question: should the State of Israel continue to encourage the construction of seawater desalination plants? Present your position on this issue and justify it.”	Essays were coded for: • Structure • Number of positions presented and their justification.

Blaum et al. (2017),	46 seventh grade students from a Midwestern middle school	A≠B Seven texts and a graph dealing with climate change and presenting different aspects.	**Essay writing** “Your task is to use this set of documents to write an essay explaining how and why recent patterns in global temperature are different from what has been observed in the past and what we can do about it. Be sure to use specific information from the documents to support your conclusions and ideas.” **Sentence verification task**	Essays were coded for: • Coverage of the seven important concepts for explaining global warming • References to the source Sentence verification task was coded for: Students rated the extent to which 18 sentences were potential connections between the texts, from inconsistent (1) to consistent (6) with the ideas in the texts.

Gil et al. (2010a)	53 undergraduates from two universities in Valencia	A≠B Seven separate texts about different aspects of climate change. One text provided neutral general information and the other texts were conflicting or convergent.	**Summary∖Argument writing** (two conditions) “Base your report on information included in the following seven texts. Use the most relevant information, and try to express yourself clearly and to elaborate the information—preferably in your own words.” **Sentence verification task** Readers judged whether 26 statements were valid or invalid inferences.	Essays were coded for idea units, and each unit was coded for degree of transformation: • Paraphrasing • Elaboration • Additions • Misconceptions • Number of sources • Number of switches between sources See also: Stahl et al. (1996); Gil et al. (2010b).

Goldman et al. (2013)	211 students in Grades 5 (*n* = 70), 6 (*n* = 90), and 7 (*n* = 51)	A+B Texts that provide complementary reasons in answer to the question: Why were the civil rights events of 1955–1965 more successful than previous civil rights events? Prior to the texts, readers received an audio and animation introduction to the inquiry question.	**Writing an essay to answer an inquiry question** “Why were the civil rights events of 1955–1965 more successful than previous civil rights events?”	Essays were coded for: • Number of essay statements that were directly copied from the texts • Number of essay statements that were paraphrases of statements from the texts • Word count • Inferences not related to synthesis Instances of prior knowledge use • Number of distortions of presented content

Griffin et al. (2012)	59 American seventh grade students	A+B Seven documents Containing convergent information about the causes of global temperature change.	**Writing an essay** “Use this set of documents to write an essay explaining how and why recent patterns in global temperature are different from what has been observed in the past.”	Essays were coded for: • Including central concepts See also: Braasch et al. (2014); Linderholm et al. (2016).
			**Sentence verification task** 18 statements that represented potential connections or inferences that could or could not be made based on the information in the document set.	Sentence verification task was coded for: • Judging whether integrative statements were valid or not

Hastings et al. (2012)	460 students in Grades 5,6,7,8	A+B Three texts, each providing different reasons for the increase in the population of Chicago from 1830–1930.	**Writing an essay to answer an inquiry question** “In 1830 there were 100 people living in Chicago. By 1930, there were three million. Why did so many people move to Chicago?”	Use of three computer programs that automatically recognize • Covering main ideas

Le Bigot and Rouet (2007)	65 students from a university in France	A+B Seven short hyper texts, about different aspects of social influence.	**Short essays** “You will have to read and understand texts about the social influence. You will then have to write a one-page (about 5–10 lines) summary based on this set of texts. The summary will have to present the main ideas expressed in these texts on the subject of social influence.”	Essays were coded for: • Length • Connectives • Transformed information • References to documents

Linderholm et al. (2014)	183 undergraduate students from the southeast United States	A+B Three expository texts on the topics of electrical circuits, batteries, and lightning. Some of the content overlapped and some was unique to each text.	**Essay writing** “Imagine that you are a teacher and have to explain how *circuits* work to your students—but you have to do so in writing. Explain (in writing) how circuits work and illustrate your explanation with examples from each of the three texts you have read. Be sure to incorporate ideas from each of the three texts to explain to your students how circuits work.”	Essays were coded for: • Paraphrases • Integration within texts • Integration across texts • Statements including novelty • Information not included in any text

Rouet et al. (1997)	11 graduate students of psychology and eight graduate students of history	A≠B Seven texts dealing with the Panama Canal and presenting two controversies. The texts included historians’ essays, official documents, participants’ accounts, and a textbook excerpt.	**Essay writing** History doctoral students expressed their opinion on the controversy.	Opinions were scored as no claim, restricted claim, or full claim • Contextualization • Sourcing • Corroboration

Stadtler et al. (2013)	100 Undergraduates From a German university	A≠B Four texts that contained two controversial issues.	**Writing an essay** “Your friend is asking you to assist her by carefully reading the materials so that you will later be able to report what you have found out. She needs your information to make a knowledge-based decision about whether to take action to lower her high cholesterol level.”	Essays were coded for: • Reporting conflicts in a two-sided or one-sided manner • References to sources See also: Stadtler et al. (2014).

Kobayashi (2007)	80 Japanese students from Shizuoka University	A≠B Six letters to the editor concerning English education in elementary school in Japan.	**Intertextual relation task** Readers were required to describe how the six text writers’ arguments were interrelated with one another.	Answers were coded for: • Describing how arguments were related to each other

Bråten et al. (2013a)	65 Norwegian tenth graders	A≠B Five texts that presented different perspectives on sun exposure and health. The first text was neutral, while the four others contained partly conflicting information.	**Three open ended questions** that required participants to consider each perspective’s claim, integrate perspectives across texts, and pit perspectives against each other.	Answers were scored for: • Main arguments • Supporting reasons • Opposing arguments • Reconciling conflicts See also: Bråten et al. (2013b); Bråten et al. (2014a); Ferguson and Bråten (2013); Strømsø et al. (2016). ^∗^Bråten et al. (2013b) used two open-ended, short-essay questions.

Cerdán and Vidal-Abarca (2008)	56 undergraduate students enrolled in a psychology program at the University of Valencia, Spain.	A+B Three texts that describe a physical phenomenon, presented on a computer screen.	**Three open ended questions** asking about a practical case in which students had to apply their new knowledge to a new situation.	Answers were coded for: • Inclusion of the relevant idea units from the text. • Number of non-consecutive readings of relevant units of information, which indicated an effort to connect and integrate the two paragraphs, was assessed by computer software.

List and Alexander (2015)	215 undergraduate students at a large mid- Atlantic university in the United States.	A≠B, A+B Library of seven digital texts, specific to each of four questions assigned, dealing with psychology or astronomy.	**One open ended question and one** **question that demanded a short** **written answer**	Answers were coded for: • Number of words • Use of evidence and elaborations • Amount of information included • Connections between details.

Merkt et al. (2017)	127 ninth graders from German Secondary schools	A≠B Eight documents about the German Emergency Law that was introduced in West Germany.	**Open ended questions** While the documents were still available, the students answered two open-ended questions. The first question required them to name commonalities and differences between the documents with regard to the attitudes reflected in the documents toward the German Emergency Law. The second question (Q2) asked for the reasons for the commonalities and differences between the documents. This task required participants to situate source information about the documents in a historical context.	Q1- • Number of commonalities and differences Q2- • Noting that the documents’ origin was the main reason for the commonalities and differences.

Beker et al. (2016)	27 Leiden University undergraduates studying education sciences or psychology	A≠B Pairs of expository texts dealing with animals, objects, persons, etc. The texts included inconsistencies, with or without explanations and elaborations.	**Oral recall of texts’ content**	Mentioning unique information in the texts

Wolfe and Goldman (2005)	44 sixth grade American students	A≠B Two opposing historical accounts of the fall of Rome, a time line, a map, and a fact list.	**Answering an integrative question orally, an interview, and think aloud** “If someone were to ask you why the Roman Empire could not defend themselves against the Barbarian invasion, what would you say to that person?”	Integrative question was coded for: • Number of reasons mentioned • The complexity of the reasoning • Integration of causes Think aloud protocols were coded for: • Paraphrasing • Elaborations • Predictions


**Table 2:** Literature review of receptive integration tasks.

Researchers	Readers	Texts	Task
Maier and Richter (2016)	39 university undergraduates in Germany	Two texts arguing for contrary positions regarding whether or not electromagnetic radiation from cell phones causes possible health risks.	**Sentence verification** Participants indicated whether 48 sentences matched the content of the texts. The sentences used were: • Paraphrases • Inferences • Distractor items Situation model strength for each text was based on the probit-transformed proportion of yes responses to inference items minus the probit-transformed proportion of yes responses to the distractor items. See also: Maier and Richter (2013, 2014); Hagen et al. (2014).

Bråten and Strømsø (2006)	75 teacher students at a college in southeast Norway	A≠B Seven texts about different aspects of attention-deficit hyperactivity disorder (ADHD) were presented either separately or as a textbook.	**Sentence verification** Readers judged whether sentences that combined information from two texts created either a valid or an invalid inference. See also: Strømsø et al. (2008); Strømsø and Bråten (2009); Bråten and Strømsø (2010, 2011); Salmerón et al. (2010); Strømsø et al. (2010); Karimi and Atai (2014); Karimi (2015).

Wiley and Voss (1999)	64 undergraduates at the University of Pittsburgh, United States	A+B Eight documents dealing with Ireland from 1800 to 1850, such as a map, brief descriptions of the Act of Union, the Act of Emancipation, etc. These were presented either on a computer as a web like environment, or as a book chapter.	**Sentence verification** Students were asked whether 10 statements that demanded inferencing were true on the basis of the information they read. **Identification task** Students were asked to indicate on a l–10 scale how similar the causes of other historical scenarios were to the texts that were read.

Davis et al. (2017)	83 students in Grades 5–7, from the southwestern United States	A≠B Three texts dealing with new classifications of plants with variations in tone and authorial credentials.	**Sentence verification** The readers judged whether 35 items were correct or not according to the texts.

Mateos et al. (2016)	476 students from two universities located in Madrid and Barcelona	A≠B Three texts on the topic of nuclear energy.	**Sentence verification** A test of 22 items in which students were asked to decide in each case “whether the idea expressed can be deduced (or not) from the information included in the texts.” The items were either statements that could be answered based on the information in one text or statements that required integrating information from at least two of the texts.

Kobayashi (2015)	44 Japanese undergraduate students	A≠B 20 pairs of texts dealing with fictitious scientific, social, or personal issues.	**Sentence verification** Readers judged whether 20 statements (one statement per text) were valid or not.

Britt and Sommer (2004)	28 undergraduates at Northern Illinois University	A≠B Two texts that described the same historical event, the United States assuming control of the Illinois Territory from Indian tribes. One text was critical of the United States government and the second was supportive.	**Time-line task** • Arranging 16 target events in the correct chronological order (Experiment 1). **Forced-choice test of integration** • A list of 20 events from which participants were to select one of two stated events that occurred next in the sequence in actual time.

Wineburg (1991)	Eight high school students and eight historians (who possessed a doctoral degree, or doctoral students) in the United States	A≠B A set of eight written and three pictorial documents that dealt with the Battle of Lexington.	**Choosing a picture that matches the content of the texts Think alouds** Readers read eight texts and were asked to choose the picture that best describes the battle, while thinking aloud. Their comments were coded for: • Descriptive statements • References to texts • Statements related to point of view, intensions, and goals (analysis) • Evaluations of sources (qualifications)


**Table 3:** Results of MTI tasks review presented by text types, tasks, assessments of products and processes.

Text types	A≠B (42), A+B = (11)					
**Tasks**	**Expressive tasks (30)**			**Receptive tasks (29)**		
	Oral questions *(3)* Open-ended questions *(8)* Essay writing *(19)*			Sentence verification *(26)* Arranging events on a time line *(2)* Matching a picture to a set of texts *(1)*		
		
**Product assessment**	***Selecting information***	**Intertextual relationships**	**Inference making**	**Selecting information**	**Intertextual relationships**	**Inference making**
		
	**Conceptual level** Covering main ideas or arguments *(13)* Supporting an argument *(13)* Inaccuracies *(3)* **Linguistic level** Copy from text *(2)* Elaborations *(7)* Paraphrasing *(5)* **Rhetoric level** Structure *(2)* References to sources *(6)* Switches between sources *(2)* Word count *(3)*	**Conceptual level** Stating a position on the issue *(6)* Rebuttals∖Reconciling conflicts *(9)* Corroboration *(7)* **Linguistic level** Connectives *(1)*	**Conceptual level** Inference making not related to synthesis *(1)* Contextualization∖Use of prior knowledge*(2)* Transformed information *(1)* Additions *(2)* Statements including novelty *(1)* Evaluation *(1)*	**Conceptual level** Inaccuracies *(4)* **Linguistic level** Judging whether paraphrased sentences are valid *(4)*	**Conceptual level** Judging whether integrative statements are valid *(24)* Choosing a picture to best match a set of historical texts *(1)* Arranging historical events on a time line	**Conceptual level** Inference making *(5)* ts *(2)*
				**Process assessment** Think aloud *(2)*	Non-consecutive readings of relevant units of information *(1)* Paraphrasing *(1)* Descriptive statements *(1)* Evaluating the various texts *(2)* Elaborations *(1)* References to the texts *(1)* Statements related to point of view, intentions, purposes and goals *(1)*


*The number in brackets states the number of studies in which each item appeared*.

## Literature Review Results

The review resulted in 50 MTI studies. Table 3 summarizes the results of the review in a table, divided by text, type of task, and assessment method. Different coding schemes were used to measure text integration, where some of the parameters were repeated across studies and others were unique. We categorized them by different levels and types (see “Method” section), listed them, and noted in brackets the number of studies in which each was used. We based our coding scheme on the aim of the review, and mainly discuss text types, tasks, and assessment rubrics.

### Participants

Multiple text integration has been examined with readers of varying age, from as young as fifth grade to undergraduate students.

### Texts

The studies cited here used two to eight expository texts, such as journal articles, arguments, textbook excerpts, etc. Some also included visual information such as graphs and pictures (e.g., Wineburg, 1991). Earlier studies used historical texts, and later studies also encompassed texts from other fields, such as biology, health, and science. Comparisons of text integration across different disciplines suggest that integration is related to conventions of the disciplines chosen. For example, inconsistencies between historical accounts can be explained by different perspectives or agendas. In contrast, inconsistencies in scientific findings would be explained by differences in methodology, artifacts, etc.

Our review did not examine sourcing, or how readers examine the credibility of sources and decide on which text to rely. The participants did not search for the documents themselves, rather the texts used in the studies were presented as credible sources and the readers did not have to decide which to trust. Usually, the texts were equally relevant to the target questions. In one exceptional study, Anmarkrud et al. (2013) used texts that varied in their relevance to the inquiry question and each text had a different weight in the integration process.

The various sets of texts had two types of relationships between them: 42 studies used texts that represented a major conflict. For example, two texts that describe a historical event: one that supports United States government actions, and another that criticizes them and supports the Indian tribes’ position (e.g., Britt and Sommer, 2004). Eleven studies used texts that presented different aspects of an issue or texts that complemented each other. For example, Goldman et al. (2013) designed three texts that each offered a possible reason for a historical event. The different reasons did not contradict each other but rather supported each other. Three studies used two research conditions, one with conflicting texts and another with contradictory texts. These were coded as both A+B, A≠B.

### Expressive Tasks

Nineteen studies used essay writing as a measure of MTI, eight studies used open ended questions, and three used oral questions. Regarding essay writing, readers typically received a specific question to answer and elaborate instructions regarding what the essay should contain. For example: “Use this set of documents to write an essay explaining how and why recent patterns in global temperature are different from what has been observed in the past” (Griffin et al., 2012).

Usually, the researchers developed a set of specific categories for coding the essays. For example, Goldman et al. (2013) coded essays for: number of essay statements that were copied directly from the texts, number of paraphrases of statements from the texts, word count, inferences not related to synthesis, and instances of prior knowledge. In other cases, essays were separated into idea units and divided into categories such as paraphrasing, elaborations, etc. (e.g., Gil et al., 2010a). Two other scoring systems used were examining whether the relevant information was included in the answer, and scoring the essay holistically (not by dividing it into units) according to the quality and quantity of arguments. This was also the common coding system for open ended questions (e.g., Bråten et al., 2013a). Other coding systems for open ended questions were coding the content according to specific categories or idea units, as explained above. The same types of coding systems were used when integration was measured with oral questions. The written products were typically assessed by two judges, disagreements were solved through discussions, and the percentage of interrater agreement was reported.

### Receptive Tasks

The common measure was the sentence verification task (e.g., Bråten and Strømsø, 2010) that was used in 26 studies. This task is comprised of phrases that combine information from different sentences in the various texts, or a combination of information from the text with information that was not written explicitly, in a way that forms either a valid or invalid inference. Bråten and Strømsø (2010) reported that the reliability for the scores on this task of sentence verification as measured by Cronbach’s alpha was 0.58. They note that although this seems to be lower than desired, data from the same study conducted in English indicated that participants who included more relevant information from most of the texts in their essays and linked information from the different texts performed much better on the intertextual inference verification task compared to participants who included less information and had difficulty integrating the texts. Another receptive measure used is multiple choice comprehension questions (Britt and Sommer, 2004; Le Bigot and Rouet, 2007). In addition, Wineburg (1991) asked participants to choose a picture that best matches the integration of the texts.

#### Comparisons of Expressive and Receptive Tasks

Comparisons between assessments were scarcely reported and more empirical research is needed in order to compare the various measures. Griffin et al. (2012) examined MTI with a written essay and also with a sentence verification task. They reported that the two measures of MTI correlated only modestly with each other, but correlated similarly with other variables. They further concluded that the two measures “reflect somewhat different aspects of multiple-documents comprehension” (Griffin et al., 2012, p. 74). In contrast, Gil et al. (2010b) applied two MTI measures, essay writing and sentence verification. They found positive intercorrelations within and across the MTI measures and presented this as support for the validity of the dependent measures.

Expressive tasks appear to have higher reliability compared to receptive tasks and they are considered to measure deeper levels of integration. In a more recent work, Bråten et al. (2014a) preferred short-essay questions over intertextual inference verification tasks that they had previously used. They explained that receptive tasks have lower reliability scores and that expressive tasks make it possible “to evaluate students’ abilities to corroborate information from different sources and reason about an issue in terms of claims and evidence concerning different perspectives” (Bråten et al., 2014a, p. 18).

### Expressive and Receptive Task Assessment

Coding schemes used to evaluate integration products were heterogeneous. Regarding the assessment of expressive tasks, we found that assessment categories that belonged to the first level of selecting information were most prevalent (56). There were 23 instances of generating intertextual relationships, and only eight examples of inference making. A different pattern was found for receptive tasks. The most prevalent level was generating intertext relationships (27) compared to selecting information (8) and inference making (5). In both expressive and receptive assessments, most coding schemes focused on the conceptual level of integration, and to a lesser extent on the rhetorical and linguistic level.

Interestingly, we found at times that a task had the potential of encouraging readers to generate new inferences. However, the assessment method did not relate to instances of inference making, but only to information selecting (e.g., Griffin et al., 2012). Therefore, it seems that the level and type of integration the reader exhibits is related to the choice of texts, tasks, and assessment categories.

#### Integration Process Assessment

Two experiments used think aloud protocols, usually in order to learn about strategies that support integration (Bråten and Strømsø, 2003; Cerdán and Vidal-Abarca, 2008). Think aloud protocols provide some insight on the cognitive processes that take place when readers integrate texts and on the strategies used. In addition, Cerdán and Vidal-Abarca (2008) used a computer software to measure non-consecutive reading of relevant units of information, which according to the authors indicate an effort to connect and integrate two paragraphs.

## Discussion

The aim of the current review was to map which types of text integration were examined in empirical research. We reviewed 50 studies and noticed meaningful differences as well as similarities between them. Regarding the texts utilized in MTI tasks, we found that they were frequently contradictory (e.g., presented different opinions). Fewer studies used complementary texts, and other types of relationships were not reported. Goldman (2015) noted that the research should develop taxonomies of intertextual relations that explain how readers process multiple texts, in order to detect these relationships and how they are related to features of texts. Goldman (2015) gave examples not only of texts that agree or disagree, but also of texts that overlap in terms of content, or texts that explain one another. Recently, Strømsø (2017) also noted that “Less is known about how models of multiple source use apply to information sources containing only overlapping, complementary, or unique information” (Strømsø, 2017, p. 22).

Regarding the tasks, one salient finding was that in all the studies we reviewed the participants were given scaffolds in the form of a specific inquiry question. The inquiry question often served as a criterion that assisted the readers in selecting the relevant information from each text and in detecting associations between the texts. Readers either had to locate conflicts between the texts or join together pieces of information. We encountered no cases where participants were given a set of texts and were required to generate a title, an inquiry question, or conclusions by themselves.

For example, students were asked about the relationship between sun exposure and health, and were given texts stating that sun exposure is dangerous and other texts reporting the benefits of sun exposure (Bråten et al., 2013a). Another possible integration task would be to present students with the same texts attached to questions asking about the relationship between the texts or about conclusions that can be derived from them. In this manner, students would be encouraged to employ higher level thinking, to generate generalizations, and to form their own categorizations.

As for assessment methods, we found variations in assessments of integration tasks that reflected different conceptualizations. First, we found differences between receptive and expressive tasks in the types of integration measures as well as in the reported reliability. Second, the various scoring systems of expressive measures gave different weight to the conceptual, rhetorical, and linguistic level. While some essay scoring systems considered the structure and coherence of the argument (e.g., Anmarkrud et al. (2013); Barzilai and Eshet-Alkalai, 2015, other scoring systems reflected the conceptual level and coded essays for including key ideas (e.g., Linderholm et al., 2016). Third, among the three integration categories that we chose to use, the salient ones were *selecting information* and *generating relationships*. The category of *inference making* that related to transforming the information and producing new information was used less often.

We argue that the type of text, task, and assessment method employed focused on the literal level of the texts. Providing readers with a specific inquiry question serves as a scaffold for generating intertext links. In addition, using conflicting texts and the assessment methods described above focus only on selecting information and creating intertext relationships. We propose that more attention should be assigned to MTI that does not include scaffolds. Assessment methods described here rarely asked students to integrate texts in a way that transforms knowledge or creates new categories. We therefore wish to suggest which types of integration are currently missing from empirical research and theoretical models.

## What is Missing from Current Research?

As stated earlier, we propose that MTI tasks should also include tasks where readers generate their own categories for integration. This involves *generalization* and *abstraction*, which we consider to be higher level processes because they build on selecting information and synthesis and require the person to create his own category relevant to the texts rather than using an existing one.

According to classical definitions of Greek philosophy (Bäck, 2014), *generalizing* is the process of reaching general conclusions or formulating principles from an array of details. For example, a person may read three texts by the same author and conclude that all the texts deal with family relationships. Generalizing is therefore a form of creating new knowledge. This knowledge is not absolute, and is subject to change if new information appears, such as a new text by the same author that deals with different issues.

Abstraction refers to the process of disregarding contingent details for the sake of reaching the essence of a certain object. An example might be recognizing a common underlying assumption across several texts, or designing a rule or a theoretical model based on several concrete situations. In these cases several features of objects are ignored in order to reveal core similarities between them.

Generalization and abstraction are common in academic settings. Scholars read articles describing specific findings and generate synthesis, organization of information, generalizations, and abstractions on a daily basis. One type of integration involves recognizing main ideas and similar themes and understanding whether the findings support or contradict each other and how they relate to previous findings. A higher level of integration would be performing generalizations and abstractions, reaching general conclusions, and identifying a common essence of the various texts. Imagine, for example, a student reading several journal articles about various variables that predict reading comprehension. The student may identify the main ideas in each text, recognize relationships between the texts, and organize the information as follows: “Accuracy and speed of word reading, as well as vocabulary knowledge, contribute to reading comprehension in primary school.” However, the student may also try to reach a higher level of integration (generalization and abstraction) and add that the type of reading comprehension assessment affects study results, or realize that reading comprehension research focuses more on simple rather than on deep reading comprehension. Other examples might be reading different works of the same writer to describe common elements in his or her work, or reading different studies and exposing similarities and differences in the underlying theoretical models.

Integration based on generalization and abstraction is common in academic contexts, and in these situations the relationships between the texts, the reading goal or task, can be different than those presented in experimental studies. Similarities between texts are more implicit, non-concrete, and are sometimes not found on the text base level but only on the situation model level. The texts might share core features that the readers need to extract, generalize, and abstract. The relationships between the texts are less structured and clear, and the reading goal may also be less specific. Instead of one correct answer, there might be different options of information integration.

We wish to incorporate the main themes from the definitions presented earlier and to suggest that MTI is a process of linking pieces of information from various texts and their sources. Links are formed on the basis of identifying similarities and differences, as well as on inference making on different levels of the text, such as the textbase and the situation model (Kintsch, 1988, 1998). MTI results in several possible types of links between texts: extracting relevant ideas, synthesis, generalization, and abstraction.

The type of integration that takes place depends on the reader, the task, the reading activity, and the context (Snow, 2002). Readers can be more or less likely to reach different types of integration. Hartman (1995) suggested that different readers integrate sets of texts differently, and identified three approaches: The *logocentric* approach refers to limiting oneself to the author’s intent. The *intertextual* approach means trying to link as much information as possible, and the *resistant* approach refers to criticizing the texts and arguing with them. Thus, certain readers will pursue higher order integration even when dealing with simple texts and tasks, and when the assignments are freer in nature, the various readers will exhibit different types of integration. In addition, research has suggested that MTI is a difficult task that often does not occur spontaneously (Rouet, 2006; Rouet and Britt, 2011). Furthermore, differences between experts and novices reading multiple texts in their field of expertise have been demonstrated (e.g., Wineburg, 1991, 1998) and substantial research supports the contribution of various aspects of epistemic thinking (Bråten and Strømsø, 2011) and other personal traits (Barzilai and Strømsø, 2018) to multiple text integration.

Regarding the texts, it is possible that texts that are closer in their contents and that have more structured and easily recognized interrelations, direct the reader to more simple synthesis such as “Text A contradicts Text B.” When the texts share similarities that are more abstract, similarities on the situation level, integration requires more effort, and has the potential of pushing the reader to higher levels of generalization and abstraction. Thus, examination of MTI with texts that hold a variety of interrelations might yield other types of synthesis and integration.

Regarding the role of the task in integration assignments, it is possible that designing different types of tasks would result in higher levels of integration. First, different types of inquiry questions might direct the reader to different levels of integration. Specific and direct inquiry questions indeed direct the reader to analysis, synthesis, and coherent organization of information (Goldman et al., 2013). However, tasks with less scaffolding have the potential of directing the reader to reach higher levels of integration. A question that is general rather than specific can also lead to a larger variety of questions.

Second, it is possible that within the context of a research design that includes a time constraint and an encounter with new texts, reaching the highest levels of integration is extremely challenging. Perhaps in more natural settings, when dealing with familiar topics with more time in hand, readers have the potential of reaching higher levels of integration.

## Conclusion

Multiple text integration is a complex concept that builds on different processes and skills and is influenced by variables related to the reader, the texts, and the reading activity (Snow, 2002; List and Alexander, 2017). In this paper we sought to map how MTI is assessed in current research. We argue that more research is needed in order to compare between text integration tasks and that current MTI research does not represent the wide variety of MTI situations. More specifically, we suggest that empirical studies have focused on integration that is scaffolded. Finally, we describe two levels of integration, which we call generalization and abstraction, that have not received research attention so far, partly due to the choice of texts, tasks, and assessment rubrics in the various studies (Goldman, 2014; Strømsø, 2017).

We believe that this review has both theoretical and practical importance. First, this work extends our understanding of the essence of integration and serves as an initial taxonomy of types and levels of integration that will eventually lead to a deeper and broader understanding of integration processes. This work is therefore important not only for multiple text research but also for single text reading research, as the concept of integration is relevant to any form of reading comprehension. On the practical level, we pointed to a lack of studies that examine the highest levels of integration common in academia, in the work of scholars and students, as they read specific findings and are required to reach general conclusions. Thus, research of integration in the form of generalization and abstraction will extend our knowledge of these processes, which could later be used to promote integration among students.

## Author Contributions

LP conceptual development, data review, and write up. TK conceptual.

## Conflict of Interest Statement

The authors declare that the research was conducted in the absence of any commercial or financial relationships that could be construed as a potential conflict of interest.
